# A Serological Survey of Antibodies to H5, H7 and H9 Avian Influenza Viruses amongst the Duck-Related Workers in Beijing, China

**DOI:** 10.1371/journal.pone.0050770

**Published:** 2012-11-30

**Authors:** Peng Yang, Chunna Ma, Weixian Shi, Shujuan Cui, Guilan Lu, Xiaomin Peng, Daitao Zhang, Yimeng Liu, Huijie Liang, Yi Zhang, Li Zhang, Holly Seale, Quanyi Wang

**Affiliations:** 1 Institute for Infectious Disease and Endemic Disease Control, Beijing Center for Disease Prevention and Control (CDC), Beijing, China; 2 School of Public Health and Community Medicine, UNSW Medicine, The University of New South Wales, Sydney, New South Wales, Australia; University of Georgia, United States of America

## Abstract

The continued spread of highly pathogenic avian influenza (HPAI) viruses of H5 and H7 subtypes and low pathogenic avian influenza (LPAI) viruses of H5, H7 and H9 subtypes in birds and the subsequent infections in humans pose an ongoing pandemic threat. It has been proposed that poultry workers are at higher risk of exposure to HPAI or LPAI viruses and subsequently infection due to their repeated exposure to chickens or domestic waterfowl. The aim of this study was to examine the seroprevalence of antibodies against H5, H7 and H9 viruses amongst duck-related workers in Beijing, China and the risk factors associated with seropositivity. In March, 2011, 1741 participants were recruited from (1) commercial duck-breeding farms; (2) private duck-breeding farms; and (3) duck-slaughtering farms. Local villagers who bred ducks in their backyards were also recruited. A survey was administered by face-to-face interview, and blood samples were collected from subjects for antibody testing against H5, H7 and H9 viruses. We found that none of the subjects were seropositive for either H5 or H7 viruses, and only 0.7% (12/1741) had antibody against H9. A statistically significant difference in H9 antibody seroprevalence existed between the various categories of workers (*P* = 0.005), with the highest figures recorded amongst the villagers (1.7%). Independent risk factors associated with seropositivity toinfection with H9 virus included less frequent disinfection of worksite (OR, 5.13 [95% CI, 1.07–24.58]; *P* = 0.041; ≤ twice monthly versus>twice monthly) and handling ducks with wounds on hands (OR, 4.13 [95% CI, 1.26–13.57]; *P* = 0.019). Whilst the risk of infection with H5, H7 and H9 viruses appears to be low among duck-related workers in Beijing, China, ongoing monitoring of infection with the H9 virus is still warranted, especially amongst villagers who breed backyard ducks to monitor for any changes.

## Introduction

The spread of highly pathogenic avian influenza (HPAI) viruses of H5 or H7 subtypes and low pathogenic avian influenza (LPAI) viruses of H5, H7 or H9 subtypes amongst birds and sporadic infection in humans continues to pose a threat to public health [1]–[10], because of the potential for a strain with pandemic potential to emerge via adaptive mutation or reassortment [11]. The 1918 pandemic begun following adaptive mutation of an avian virus, and the pandemics of 1957 and 1968 were the result of genetic reassortment of viruses from human and avian sources [12]–[14]. While the last pandemic originated from a swine-origin influenza virus (S-OIV) [15], the threat brought by avian influenza viruses continues.

As of September, 2012, a total of 608 human cases of HPAI H5N1 were reported globally, with a case-fatality ratio of 59.0% (359 fatal cases) [16]. In addition, human cases infected with H7 or H9 viruses were also sporadically reported in a couple of countries. In 1996, a woman developed conjunctivitis after a piece of straw had entered her eye while cleaning her duck house, with LPAI H7N7 virus isolated from her conjunctiva specimen [3]. In 2003, 89 people were confirmed as being infected with HPAI H7N7 in the Netherlands with evidence of limited person to person transmission, which was associated with an outbreak of HPAI H7N7 in chickens in over 200 farms [4], [5]. In 2007, four cases infected with LPAI H7N2 were identified after an outbreak of LPAI H7N2 in chickens on a small farm in north Wales in the United Kingdom [6]. Eight cases were confirmed as being infected with LPAI H9N2 from mainland and Hong Kong, China in 1998–1999, of which three had contact with live chickens before illness onset [7]–[9]. In 2003, a five-year-old child infected with LPAI H9N2 was reported in Hong Kong, China. However, the source of infection was unknown [10].

To date most studies, especially those on risk factors of infection, have generally focused on H5 virus rather than H7 or H9 viruses [17]–[22]. Given the distinct possibility of human infection with H7 or H9 viruses and the minimal human-to-human transmission of avian-adapted viruses, it is important that the research focus shift accordingly. Previous studies have demonstrated that both direct and indirect exposure to infected live/dead poultry, including chickens or domestic waterfowl, plays a very important role in the transmission of HPAI or LPAI viruses to human [3]–[7], [17], [20], [22]. Poultry workers are therefore considered to be at highest risk of infection with HPAI or LPAI viruses because of their frequent exposure to chickens or domestic waterfowl. In addition, as China is considered to be an an influenza epicenter [23], ongoing monitoring of infection from HPAI or LPAI viruses in China is warranted.

In this study, we conducted a serological survey of antibodies against H5, H7 and H9 viruses amongst duck-related workers in Beijing, China to examine previous infection with these viruses and associated risk factors in this population.

## Materials and Methods

### Ethics Statement

This study was approved by the institutional review board and human research ethics committee of Beijing Center for Disease Prevention and Control (CDC).

### Subjects and Survey Design

This study was conducted in Beijing, China in March, 2011. In Beijing, 14 of the 18 districts are classified as having some form of duck-related industry. 6/14 districts were randomly selected for inclusion in the study. Workers from commercial and private duck-breeding farms and slaughtering sites were invited to participate in this study. Local villagers involved with breeding ducks in their backyards were also recruited. A duck-slaughtering site refers to a place where ducks are killed, phlebotomized, plucked, the heads/viscera removed, and processed ducks are cleaned and packaged. Workers were excluded if they were employed in a position in which exposure to ducks was limited such as those in administrative roles. From the selected six districts, all identified workers who were employed within the sector and households involved with breeding ducks in their backyards were invited to participate in this study. From each household, one family member who was deemed to have the closest contact with ducks was recruited in this study.

After obtaining written informed consent from the participants, a questionnaire was administered via a face-to-face interview conducted by trained staffs, and blood samples were collected for antibody testing against H5, H7 and H9 viruses.

### Survey Contents

The survey included questions on employment, demographics (sex, age, and education background), presence of any underlying disease, smoking, and alcohol intake. Other items included duration of exposure to ducks, farming practices (e.g. breeding patterns, duck breeds), avian influenza vaccination status of the ducks, exposure of ducks to other species of birds, disinfection of worksite, personal protective equipment use, handling ducks with wounds on hands and contact with sick or dead ducks, etc.

### Laboratory Testing

Serum samples were pretreated and assayed by hemagglutination-inhibition (HI) assay, as previously described [24]. One volume of serum was treated with four volumes of receptor-destroying enzyme (RDE) at 37°C for 18 hours, and was then incubated at 56°C for 30 minutes, followed by absorption with horse erythrocytes. The titration of 1∶10 was first prepared for each pre-treated serum sample to test for specific antibodies against H5, H7 and H9 virus antigens using 1% horse erythrocytes. H5, H7 and H9 virus antigens employed for HI assay were A/Chicken/Anhui/01/2005 (HPAI H5N1), A/Chicken/Hebei/02/2007(LPAI H7N2) and A/Chicken/Shanghai/10/1999 (LPAI H9N2), provided by Qingdao YEBIO Bio-engineering Co.,Ltd, China. The serum samples with 1∶10 titer that were able to inhibit virus-induced hemagglutination were then diluted into eight titrations (1∶10, 1∶20, 1∶40, 1∶80, 1∶160, 1∶320, 1∶640 and 1∶1280) for the HI assay. The HI titer was calculated as the reciprocal of the highest dilution of serum that inhibited virus-induced hemagglutination of the horse erythrocytes. A titer value of ≥1∶40 was regarded as positive, i.e. previous infection [25], [26].

### Statistical Analysis

Data were entered in duplicate using EpiData Software, and was analyzed using SPSS16.0 statistical package (SPSS Inc., Chicago, IL, USA). We estimated the seroprevalence rates of antibodies against H5, H7 and H9 viruses among various types of subjects. Seroprevalence rates were compared between subgroups using Pearson χ^2^ test. Seropositive (HI titer≥1∶40) and seronegative (HI titer<1∶40) groups were compared to identify potential risk factors associated with seropositivity to H5, H7 or H9 viruses (previous infection), and univariate and multivariate unconditional logistic regression analysis were conducted to determine risk factors. The variables with *P*<0.10 in univariate analysis were included in multivariate analysis. Backward logistic regression was conducted with a probability of removal set at 0.1, i.e. all variables with p<0.10 were left in the final model. All the tests were 2-sided, and statistical significance was defined as *P*<0.05.

## Results

### Characteristics of Subjects

A total of 1741 subjects, with the median of age of 44 years (range: 14–71 years), were involved in this study, including 313/374 (response rate: 83.7%) participants from the commercial duck-breeding farms, 261/286 (response rate: 91.3%) from private duck-breeding farms, 562/620 (response rate: 90.6%) from farms which slaughtered ducks, and 605/710 (response rate: 85.2%) villagers who bred backyard ducks. The participant demographic information is summarized in Table 1.

**Table 1:** Baseline characteristics of study participants (n = 1741).

Characteristic	Frequency (% of total)
Participant category	
Commercial duck-breeders	313 (18.0)
Private duck-breeders	261 (15.0)
Villagers breeding backyard ducks	605 (34.7)
Duck-slaughtering workers	562 (32.3)
Sex	
Male	759 (43.6)
Female	982 (56.4)
Age group	
≤50 years	1191 (68.4)
>50 years	550 (31.6)
Ethnic group	
Chinese Han	1637 (94.0)
Chinese Minority Groups	104 (6.0)
Education background	
>Primary school	972 (55.8)
≤Primary school	769 (44.2)
Marital status	
Not married	207 (11.9)
Married	1515 (87.0)
Divorced	6 (0.3)
Widowed	13 (0.7)
Having underlying disease	
No	1592 (91.4)
Yes	149 (8.6)
Currently smoker	
No	1266 (72.7)
Yes	475 (27.3)
Currently drinker	
No	1208 (69.4)
Yes	533 (30.6)

### Antibody Seroprevalence Against H5, H7 or H9 Viruses

In this study, none of the subjects were seropositive for either H5 or H7 antibodies, and only twelve (0.7%, 12/1741) had antibody against H9, among which four had a titer of 1∶80 and eight with a titer of 1∶40. Ten of the villagers were seropositive to H9 (1.7%, 10/605), but none of the commercial duck breeding farmers were positive. There was a statistically significant difference in seroprevalence of antibody against H9 between the various working categories (*P* = 0.005). No statistically significant difference was found for H9 seroprevalence between the sexes (*P* = 0.892), but a difference was found between age groups (*P* = 0.021), with the higher seroprevalence recorded for subjects above 50 years (1.5%, 8/550). The breakdown by group is shown in Table 2.

**Table 2:** Seroprevalences of antibodies to H5, H7 and H9 viruses in duck-related workers in the serological survey in Beijing, China.

Characteristic	Subject number	H5 virus	H7 virus	H9 virus	*P* value^a^
Participant category					
Commercial duck-breeders	313	0 (0)	0 (0)	0 (0)	0.005^b^
Private duck-breeders	261	0 (0)	0 (0)	1 (0.4)	
Villagers breeding backyard ducks	605	0 (0)	0 (0)	10 (1.7)	
Duck-slaughtering workers	562	0 (0)	0 (0)	1 (0.2)	
Sex					
Male	759	0 (0)	0 (0)	5 (0.7)	0.892^b^
Female	982	0 (0)	0 (0)	7 (0.7)	
Age group					
≤50 years	1191	0 (0)	0 (0)	4 (0.3)	0.021^c^
>50 years	550	0 (0)	0 (0)	8 (1.5)	
Total	1741	0 (0)	0 (0)	12 (0.7)	

**NOTE.** Data are seropostive no (%), unless otherwise indicated.

a Comparison of H9 antibody status.

b Compared by Pearson χ^2^ test.

c Compared by Pearson χ^2^ test with continuity correction.

### Risk Factors Associated with Seropositivity for Antibodies to H9 Virus in Duck-related Workers

In univariate analysis, the following factors were found to be significantly associated with seropositivity for antibodies to H9 virus (Table 3): older age (odds ratio [OR], 4.38 [95% confidence interval [CI], 1.31–14.61]; *P* = 0.016; >50 years versus ≤50 years), fewer years of education (OR, 3.83 [95% CI, 1.03–14.18]; *P* = 0.045; ≤primary school versus above primary school), exposure to ducks ranging on land and river (OR, 3.91 [95% CI, 1.17–13.04]; *P* = 0.026; versus ranging only on land), exposure to layer ducks (OR, 6.36 [95% CI, 1.72–23.59]; *P* = 0.006; versus broiler ducks), exposure to ducks in contact with other birds (OR, 3.92 [95% CI, 1.24–12.41]; *P* = 0.020), less frequent disinfection of worksite (OR, 7.48 [95% CI, 1.63–34.22]; *P* = 0.010; ≤ twice monthly versus>twice monthly), noncompliance with mask use (OR, 8.06 [95% CI, 1.04–62.55]; *P* = 0.046) and handling ducks with wounds on hands (OR, 6.33 [95% CI, 2.00–20.09]; *P* = 0.002).

**Table 3:** Univariate analysis for risk factors associated with seropositivity for antibodies to H9 virus amongst duck-related workers, Beijing, China.

Factors	Seropositivity (n = 12)	Seronegativity (n = 1729)	OR (95% CI)	*P* value*
Sex				
Male	5 (41.7)	754 (43.6)	Reference	
Female	7 (58.3)	975 (56.4)	1.08 (0.34–3.43)	0.892
Age group				
≤50 years	4 (33.3)	1187 (68.7)	**Reference**	**0.016**
>50 years	8 (66.7)	542 (31.3)	**4.38 (1.31–14.61)**	
Education background				
>Primary school	3 (25.0)	969 (56.0)	**Reference**	
≤Primary school	9 (75.0)	760 (44.0)	**3.83 (1.03–14.18)**	**0.045**
Having underlying disease				
No	10 (83.3)	1582 (91.5)	Reference	
Yes	2 (16.7)	147 (8.5)	2.15 (0.47–9.92)	0.325
Currently smoker				
No	9 (75.0)	1257 (72.7)	Reference	
Yes	3 (25.0)	472 (27.3)	0.89 (0.24–3.29)	0.859
Currently drinker				
No	7 (58.3)	1201 (69.5)	Reference	
Yes	5 (41.7)	528 (30.5)	1.63 (0.51–5.14)	0.409
Years of exposure				
≤5 years	9 (75.0)	1259 (72.8)	Reference	
>5 years	3 (25.0)	470 (27.2)	0.89 (0.24–3.31)	0.866
Breeding pattern of ducks				
Ranging only on land	4 (33.3)	1144 (66.2)	**Reference**	**0.026**
Ranging on land and river	8 (66.7)	585 (33.8)	**3.91 (1.17–13.04)**	
Type of ducks				
Broiler ducks	3 (25.0)	1175 (68.0)	**Reference**	**0.006**
Layer ducks	9 (75.0)	554 (32.0)	**6.36 (1.72–23.59)**	
Avian influenza vaccination in ducks				
Yes	11 (91.7)	1314 (76.0)	Reference	
No	1 (8.3)	415 (24.0)	0.29 (0.04–2.24)	0.234
Ducks in contact with other birds				
No	5 (41.7)	1274 (73.7)	**Reference**	
Yes	7 (58.3)	455 (26.3)	**3.92 (1.24–12.41)**	**0.020**
Frequency of disinfection of worksite				
>twice monthly	2 (16.7)	1036 (59.9)	**Reference**	**0.010**
≤ twice monthly	10 (83.3)	693 (40.1)	**7.48 (1.63–34.22)**	
Mask use				
Yes	1 (8.3)	731 (42.3)	**Reference**	
No	11 (91.7)	998 (57.7)	**8.06 (1.04–62.55)**	**0.046**
Glove use				
Yes	3 (25.0)	815 (47.1)	Reference	
No	9 (75.0)	914 (52.9)	2.68 (0.72–9.92)	0.141
Handling ducks with wounds on hands				
No	5 (41.7)	1416 (81.9)	**Reference**	
Yes	7 (58.3)	313 (18.1)	**6.33 (2.00–20.09)**	**0.002**
Occurrence of sick/dead ducks at worksite				
No	10 (83.3)	1454 (84.1)	Reference	
Yes	2 (16.7)	275 (15.9)	1.06 (0.23–4.85)	0.943
Close contact with sick/dead ducks				
No	11 (91.7)	1498 (86.6)	Reference	
Yes	1 (8.3)	231 (13.4)	0.59 (0.08–4.59)	0.614
Close contact with other animals				
No	9 (75.0)	917 (53.0)	Reference	
Yes	3 (25.0)	812 (47.0)	0.38 (0.10–1.40)	0.144
Exposure to birds outside of work setting*				
No	12 (100)	1700 (98.3)	Reference	1.000
Yes	0 (0)	29 (1.7)	NA	

**NOTE.** Data are frequency (%) of subjects, unless otherwise indicated. Univariate unconditional logistic regression was employed to compare frequencies of exposure between seropositive group and seronegative group. OR, odd ratio; CI, confidence interval; NA, not available. Boldface indicates *P*<0.1.

* Fisher’s exact test was used because data distribution could not be analyzed by logistic regression.

In multivariate analysis, significant independent risk factors included less frequent disinfection of worksite (OR, 5.13 [95% CI, 1.07–24.58]; *P* = 0.041; ≤ twice monthly versus>twice monthly) and handling ducks with wounds on hands (OR, 4.13 [95% CI, 1.26–13.57]; *P* = 0.019) (Table 4).

**Table 4:** Multivariate analysis for risk factors associated with seropositivity for antibodies to H9 virus amongst duck-related workers, Beijing, China.

Factors	OR (95% CI)	*P* value
Frequency of disinfection of worksite		
>twice monthly	Reference	
≤ twice monthly	5.13 (1.07–24.58)	0.041
Handling ducks with wounds on hands		
No	Reference	
Yes	4.13 (1.26–13.57)	0.019

**NOTE.** Those variables with *P*<0.1 in univariate analysis were included in multivariate unconditional logistic regression analysis. OR, odd ratio; CI, confidence interval.

## Discussion

In this study, none of the enrolled workers were seropositive for either H5 or H7 antibodies, but 0.7% had antibody against H9 virus. This finding indicates that the risk of infection with H5, H7 and H9 viruses appears to be low among duck-related workers in Beijing, China, but the risk of infection with H9 virus is slightly elevated in comparison to the other subtypes.

Although HPAI H5N1 virus continues to circulate in chickens and domestic waterfowl in China [27], human infection with HPAI H5N1 virus so far has been rare, with only 43 human cases as of the end of September, 2012 [16]. Low rates of subclinical infection in poultry (chickens of domestic waterfowl) workers have previously been documented in a number of Chinese studies [28], [29]. In addition, studies conducted in other countries have also reported low frequency of transmission of this virus to poultry (chickens of domestic waterfowl) workers [30]–[32]. These reports and our findings indicate that there continues to be a strong host specificity of infection with the H5 virus.

In agreement with a previous study from Northern China, we did not find any subjects who were seropositive to the H7 virus [33]. In comparison, previous European studies have documented higher frequencies of human infection with the H7 viruses [3]–[6]. This correlates well to the surveillance studies of H7 viruses in birds which have documented persistent epidemics of the virus in birds in many European countries [1], but not in China.

In this study, ten villagers and two farm workers were seropositive to H9 antibody. Previously, a study from Guangzhou in southern China also found that the seroprevalence of antibody against H9 virus (4.5%) in poultry workers was much higher than that against H5 virus (0.2%) [29]. In addition, a study conducted in northern China found that 1.5% (12/783) of villagers breeding backyard poultry (chickens or domestic waterfowl) were seropositive to H9 virus, but identified no persons seropositive to H7 virus [33]. Our findings, along with those from previous studies support the notion that there is relatively higher risk from H9 virus in China, in comparison to H5 and H7 subtypes.

In the present study, the seroprevalence of antibodies against H9 virus amongst villagers breeding ducks in their backyard was significantly greater than participants from other categories, which was supported by a previous work [33]. This difference may be attributed to two reasons as follows: firstly, people raising ducks in their backyards have closer and longer periods of exposure to ducks, and this may put them at increased risk for exposure to viruses; secondly, people who work with ducks in their job are more likely to use personal protective equipment like masks and gloves than people raising ducks in their backyards.

In this study, the seroprevalence of antibodies to H9 virus amongst participants above 50 years was significantly higher than those less than 50 years. As there is no report about the change of susceptibility in human of the H9 viruses that have circulated in China in recent years, we consider that this difference by age may be attributed to a longer period of exposure to ducks in older participants.

Participants who reported that the worksite was infrequently disinfected were more likely to be identified as seropositivity for antibodies to H9 virus in our study. In corroboration are the results from a Korean study that found an increased risk of seropositivity against H9 virus in chickens associated with less frequent cleaning with disinfectants [34]. We also found that contact with ducks while having a hand wound was also a risk factor for previous infection with H9 virus amongst the duck workers. Given this finding, glove should be recommended for workers with wounds on hands to use.

It has been reported that handling healthy, sick and dead chickens or domestic waterfowl was the predominant means of human infection with HPAI H5N1, HPAI/LPAI H7 and LPAI H9N2 viruses [2]–[7]. In addition, for patients infected with HPAI H5N1, contact with virus-contaminated fomites followed by self-inoculation of the respiratory tract or inhalation of aerosolized infectious excreta was also plausible transmission route [2]. Therefore, mask or glove use may theoretically provide protection against H5, H7 and H9 viruses for persons having frequent contact with chickens or domestic waterfowl. In the present study, no mask use was a significant risk factor for previous infection with H9 virus in univariate analysis, but not in multivariate analysis; no glove use was not a significant risk factor either in univariate analysis or in multivariate analysis. Due to the small number of persons seropositive to H5, H7 and H9 viruses, this study may be underpowered to assess the effect of mask or glove use.

The LPAI H9N2 virus strain used in our HI assay, belonging to the BJ94-like lineage, was isolated in 1999, and it was the diagnostic antigen for detecting antibodies to H9 virus recommended by the agricultural authorities in China. Although H9N2 viruses circulating in China had experienced genetic and antigenic changes since 1999, it was found that similar sequence and obvious cross-reactivity in HI assay existed between H9N2 viruses isolated around 1999 and those isolated in the recent years, and all these viruses still belonged to BJ94-like lineage [35], [36]. In addition, as most of patients infected with LPAI H9N2 virus in China were also found around 1999 [7]–[10], using the virus isolated at that time for HI assay may have higher efficiency to detect human infection with H9 virus crossing the species barrier. In addition, although Beijing and Shanghai are located in different regions of China, there were no distinct regional differences found in LPAI H9N2 viruses circulating in China previously [35], [36]. Based on the above-mentioned reasons, we selected A/Chicken/Shanghai/10/1999 (LPAI H9N2) strain used in the HI assay for our study.

In this study, we applied HI assay using horse erythrocytes to detect human sera for antibodies to H5, H7 and H9 viruses. HI assay using horse erythrocytes has high sensitivity and specificity in detecting human antibodies against avian-specific influenza viruses [24], [26]. In comparison with HI assay using chicken or turkey erythrocytes, HI assay using horse erythrocytes has increased sensitivity, which may be explained by the fact that horse erythrocytes express a higher proportion of sialic acid containing N-acetylneuraminic acid α2,3-galactose (SAα2,3Gal) linkages which avian-specific influenza viruses preferentially bind [37]–[39].

The study population in this study was the people related to domestic ducks. However, in fact the people frequently exposed to wild ducks should be concerned about as well because wild birds are thought to form the reservoir of influenza A viruses in nature [40]. It was ever reported by Gill *et al* that of 39 duck hunters and 68 wildlife professionals in the US, three had previous infection with H11N9 virus [41]. In addition, as the worksites of the participants in our study were mainly located in rural areas, they also had the opportunity to contact wild birds and be exposed to wild bird-origin virus strains. The antigens used in this study are the diagnostic antigens recommended by agricultural authorities in China for testing antibodies against viruses in poultry (chickens or domestic waterfowl), thus we are not sure if these antigens could be used for detecting antibodies elicited by wild bird-origin strains. However, considering the similarity between wild bird-origin strains and poultry-origin strains [42]–[44], we think antigenic cross-reactivity in HI assay may exist between antigens used in this study and wild bird-origin strains.

This study has several limitations. Firstly, information regarding the exposures of participants to ducks was all based on self-report, and this study is therefore subject to recall bias. Sencondly, we only detected twelve subjects with antibody against H9 virus; therefore, this study was probably underpowered as the result of the small sample size to detect other potentially significant risk factors for previous infection. Thirdly, there could be some risk factors that were not taken into account in this study. It was a limitation that we could not assess the effect of visiting live poultry markets because selling live poultry in food markets had been banned in Beijing since 2005.

In summary, the risk of infection with H5, H7 and H9 viruses appears to be low among duck-related workers in Beijing, China, but closer monitoring of infection with the H9 virus should be warranted, especially amongst villagers breeding ducks in their backyards. Less frequent disinfection of the worksites and having contact with ducks while having hand wounds were both independent risk factors for previous infection with H9 virus amongst the duck workers.
